# Biomineralize Mitochondria in Metal‐Organic Frameworks to Promote Mitochondria Transplantation From Non‐Tumorigenic Cells Into Cancer Cells

**DOI:** 10.1002/smmd.134

**Published:** 2025-02-26

**Authors:** Jun‐Nian Zhou, Chang Liu, Yonghui Wang, Yong Guo, Xiao‐Yu Xu, Elina Vuorimaa‐Laukkanen, Oliver Koivisto, Anne M. Filppula, Jiangbin Ye, Hongbo Zhang

**Affiliations:** ^1^ Pharmaceutical Sciences Laboratory Faculty of Science and Engineering Åbo Akademi University Turku Finland; ^2^ Turku Bioscience Centre University of Turku and Åbo Akademi University Turku Finland; ^3^ Beijing Institute of Radiation Medicine Beijing China; ^4^ Department of Endocrinology Key Laboratory of National Health & Family Planning Commission for Male Reproductive Health National Research Institute for Family Planning Beijing China; ^5^ Chemistry and Advanced Materials Faculty of Engineering and Natural Sciences Tampere University Tampere Finland; ^6^ Department of Radiation Oncology Stanford University School of Medicine Stanford California USA

**Keywords:** biomineralization, cancer cells, metal‐organic frameworks, mitochondria, mitochondria transplantation

## Abstract

Mitochondria are crucial to cellular physiology, and growing evidence highlights the significant impact of mitochondrial dysfunction in diabetes, aging, neurodegenerative disorders, and cancers. Therefore, mitochondrial transplantation shows great potential for therapeutic use in treating these diseases. However, transplantation process is notably challenging due to very low efficiency and rapid loss of bioactivity post‐isolation, leading to poor reproducibility and reliability. In this study, we develop a novel strategy to form a nanometer‐thick protective shell around isolated mitochondria using Metal‐Organic Frameworks (MOFs) through biomineralization. Our findings demonstrate that this encapsulation method effectively maintains mitochondria bioactivity for at least 4 weeks at room temperature. Furthermore, the efficiency of intracellular delivery of mitochondria is significantly enhanced through the surface functionalization of MOFs with polyethyleneimine (PEI) and the cell‐penetrating peptide Tat. The successful delivery of mitochondria isolated from non‐tumorigenic cells into cancer cells results in notable tumor‐suppressive effects. Taken together, our technology represents a significant advancement in mitochondria research, particularly on understanding their role in cancer. It also lays the groundwork for utilizing mitochondria as therapeutic agents in cancer treatment.

## Graphical abstract

Our results show that a nanometer‐thick protective shell on the freshly isolated mitochondria with ZIF‐8 through biomineralization can effectively maintain their bioactivity at room temperature, resistant to extreme external environments. This encapsulation can be better modified by introducing PEI and Tat to facilitate them enter the recipient cancer cells to induce significant tumor‐suppressive effects.

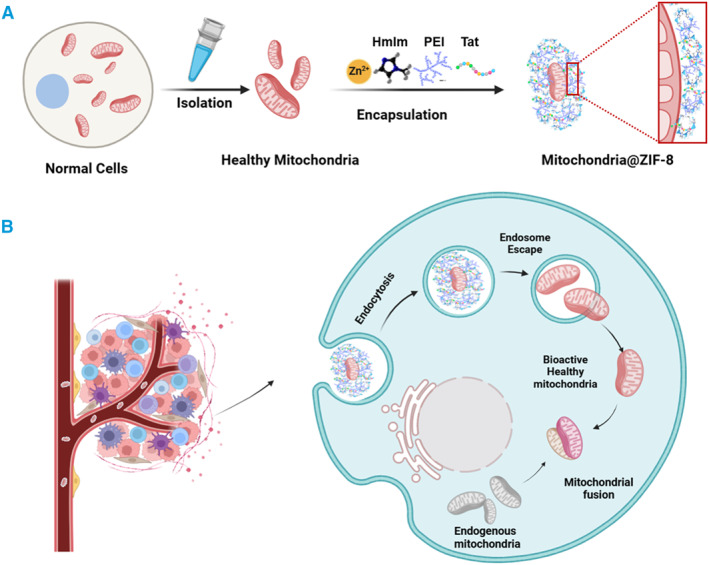

1

**Summary**

- Creating a protective shell of nanometer thickness on isolated mitochondria using Metal‐Organic Frameworks (MOFs) through biomineralization presents a promising strategy for mitochondria transplantation.
- The ZIF‐8 encapsulation technique successfully maintains mitochondria bioactivity for minimum of 4 weeks at room temperature.
- The effective delivery of ZIF‐8‐coated mitochondria, sourced from non‐tumorigenic cells, into cancer cells results in notable tumor‐suppressive effects.

**Abbreviations**
ΔΨm: mitochondrial membrane potential
CLSM: confocal laser scanning microscopy
DAPI: 4′, 6‐diamidino‐2‐phenylindole
FT‐IR: fourier transform infrared spectrometer
HmIm: 2‐methylimidazole
MIT: mitochondria
MOF: metal‐organic framework
MVG: mitoview Green
NPs: nanoparticles
PEI: polyethylenimine
RIPA: radio immunoprecipitation assay
RT: room temperature
Tat: cell‐penetrating peptide tat
TEM: transmission electron microscopy
TMRM: tetramethylrhodamine, methyl ester
ZIF‐8: zeolite imidazole framework‐8

## 2 Introduction

Mitochondria (MIT) are semi‐autonomous membrane‐bound organelles essential for various cellular functions, including nutrient biosynthesis, apoptosis, signal transduction, bioenergy metabolism [1], and epigenetic regulation and differentiation [2]. All human cells except red blood cells, contain one or more mitochondria, and sometimes even several thousand, ranging in size from approximately 500–1000 nm. Mitochondria consist of numerous bioelectric units that generate energy in the form of an array [3]. Mitochondrial dysfunctions, such as oxidative phosphorylation damage, inhibitions in the electron transport chain, apoptosis inhibition, autophagy disorders, promotion of immune escape, and signal pathway aberrations, are linked to various diseases, including diabetes, aging, neurodegenerative disorders, and cancers [4]. Therefore, mitochondria are critical targets for the treatment of a wide range of diseases and conditions.

Mitochondria are dynamic organelles that continually undergo fusion and fission in regulated ways. Currently, research focuses on developing drugs and small molecular compounds to target and regulate mitochondria's functions for cancer treatment [5, 6]. For examples, indocyanine derivative IR‐780 [7] and IR‐Pyr [8], BODIPY‐TPA (BTPA) [9] and the cyclometalated Ir (III) photosensitizer Ir‐TPE [10] have recently emerged as effective mitochondrial‐targeted fluorescent probes and therapeutic agents, particularly in cancer photodynamic therapy. Nanotechnology has led to innovative mitochondrial‐targeting cancer strategies including mitochondria‐targeted chimeric peptide‐based nanoparticle (M‐ChiP) [11], mitochondria‐targeted RNAi nanoparticle (NP) platforms [12], and nanoprodrugs delivered to mitochondria via a triphenylphosphine group (TPP^+^) [13]. In a randomized phase 0/I trial, the mitochondrial inhibitor ME‐344 combined with bevacizumab demonstrated significant antitumor activity in HER2‐negative breast cancer [14]. However, these drugs cannot fully restore mitochondrial function. Comprehensive investigations focusing on the entire mitochondria are critical to understanding their role in tumors [3]. Moreover, recent studies have revealed that mitochondrial transfer occurs between different cells [15]. Consequently, a more effective treatment for mitochondria‐related diseases has been proposed: transplanting whole mitochondria from healthy cells into diseased ones [16, 17, 18, 19]. Nonetheless, mitochondrial transplantation therapies face significant challenges: mitochondria lose bioactivity within hours after isolation from healthy cells using current techniques. Additionally, these therapies are inefficient due to the low uptake rate of mitochondria and the disruption of their structure and bioactivity during delivery process before endocytosis into target cells. Therefore, advancing mitochondrial delivery technologies is crucial for promoting the large‐scale biomedical application of mitochondria.

Metal‐organic frameworks (MOFs) are type of porous material characterized by a periodic network structure formed through the coordination self‐assembly of metal ions or clusters with organic ligands. MOF‐based biomineralization is an advanced bioencapsulation technology inspired by natural biomineralization processes. It enables the formation of a protective MOF layer directly on biomolecules and other cargo regardless of their size or surface properties [20]. By selecting suitable combinations of metal ions and organic ligands, highly biocompatible MOFs can be synthesized using a straightforward and rapid process in aqueous solution at room temperature (RT). Most important, the MOFs possess tunable molecular‐level pores that allow for basic ion and oxygen exchange while securely immobilizing the cargo's structure. This prevents the penetration by digestive enzymes, thereby safeguarding the cargo from biodegradation [21, 22]. Owing to their advanced functionalities and excellent biocompatibility, MOFs are increasingly being explored for cancer treatment. The Zeolite Imidazole Framework‐8 (ZIF‐8), formed by the coordination of Zn^2+^ ions and 2‐methylimidazole (HmIm), boasts a large surface area, exceptional chemical and thermal stability, convenient synthesis, and controllable size. Its biomimetic mineralization synthesis at RT in aqueous solution is advantageous for its biological applications, including the encapsulation and transportation of functional and active biomacromolecular substances [23, 24]. In prior studies, we have successfully encapsulated various biomolecules within MOFs, including chemical drugs, nucleic acid, anti‐PD‐L1 antibodies, and CRISPR/Cas9 riboproteins [25, 26, 27, 28, 29, 30, 31]. This proof‐of‐concept work is anticipated to advance the ZIF‐8‐based biomineralization technique involving mitochondria, paving the way for large‐scale biomedical applications. These applications include the efficient delivery of entire mitochondria into cancer cells, thereby having a significant therapeutic impact.

Here, we developed a highly facile aqueous synthesis technique that facilitates the encapsulation and protection of mitochondria with MOFs. This method preserves mitochondrial bioactivity over extended periods post‐isolation and significantly enhances targeted intracellular delivery efficiency. In the context of developing novel therapies, we provide proof‐of‐concept data demonstrating that mitochondria encapsulated from non‐tumorigenic cells can be released in a pH‐specific manner within cancer cells, resulting in pronounced tumor‐suppressing effects. This approach could serve as an essential tool for mitochondria transplantation‐based therapy by ensuring sustained bioactivity and high‐efficiency delivery. Consequently, it provides ample time to manipulate and modify numerous mitochondria in vitro for prospective clinical applications targeting mitochondria‐associated diseases.

## 3 Results

### 3.1 Loading and Characterization of MIT@ZIF‐8 Nanostructures

**FIGURE 1:**
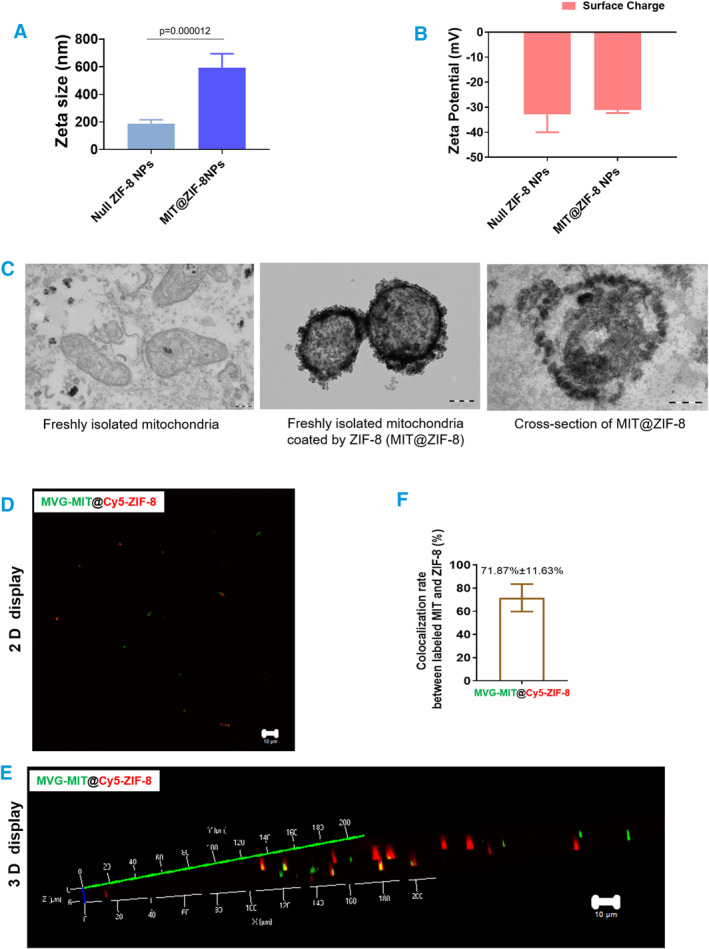
Generation and characterization of MIT@ZIF‐8 nanostructures. (A) Zeta size analysis between two groups: those incorporating with mitochondria (MIT@ZIF‐8 NPs) and those without (null@ZIF‐8 NPs). (B) Zeta potential analysis of NPs with mitochondria (MIT@ZIF‐8 NPs) or without (null@ZIF‐8 NPs). (C) Representative TEM images displaying freshly isolated mitochondria, freshly isolated mitochondria coated with ZIF‐8 (MIT@ZIF‐8) and a cross‐section of MIT@ZIF‐8. Scale bars, 200 nm. (D, E) Two‐dimensional (2D) and three‐dimensional (3D) displays of ZIF‐8 NPs using a confocal laser scanning microscope (CLSM). Scale bars, 10 μm. (F) Colocalization rate analysis between MVG‐labeled mitochondria (MIT) and Cy5‐labeled ZIF‐8.

In previous studies [25, 26, 27, 28, 29, 30, 31], we successfully encapsulated various biomolecules within ZIF‐8. Here, we explored the potential for encapsulating freshly isolated mitochondria in ZIF‐8. We developed a one‐pot synthesis method to swiftly mix and react freshly isolated mitochondria (Supporting Information S1: Figures S1, S2) with Zn^2+^ ions and HmIm at RT, generating white nanocomposites within 5 min (Supporting Information S1: Figure S3). To maintain mitochondrial enzyme activity, we used a 0.9% NaCl solution instead of PBS as the basic buffer [32, 33] (Supporting Information S1: Figure S4). Compared to null ZIF‐8 NPs, the size of MIT@ZIF‐8 was significantly larger post‐encapsulation (*p* = 0.000012) (Figure 1A), without any significant difference in zeta potential between the two groups (*p* > 0.05) (Figure 1B). Notably, a ZIF‐8 layer visibly formed atop the mitochondria in aqueous solution at RT (MIT@ZIF‐8, Figure 1C). Moreover, cut‐section analysis of the nanocomposites revealed distinct inclusion structures (Figure 1C), indicating successful encapsulation of the isolated mitochondria within the ZIF‐8 layer. Next, to evaluate the encapsulation efficiency of this process, we used Mitoview Green (MVG) dyes to stain the mitochondria of living cells (Supporting Information S1: Figure S5). After staining, we isolated the mitochondria (Supporting Information S1: Figure S6) to obtain MVG‐labeled active mitochondria, after which we performed the ZIF‐8 encapsulation. Concurrently, we added Cy5 dye to the reaction mixture to achieve Cy5‐labeled ZIF‐8 nanolayers. The resulting bicolor‐labeled NPs were imaged using confocal laser scanning microscopy (CLSM) both in 2D (Figure 1D) and 3D display (Figure 1E). We calculated the ratio of the bicolor‐labeled NPs (i.e., MIT‐MVG@ZIF‐8‐Cy5) within the total ZIF‐8‐Cy5 NPs (MIT‐MVG@ZIF‐8‐Cy5 and null@ZIF‐8‐Cy5) to assess the mitochondrial encapsulation efficiency (%). In conclusion, our findings demonstrate that freshly isolated mitochondria can be successfully coated with ZIF‐8, achieving a ZIF‐8 coating efficiency of approximately 72% (Figure 1F).

### 3.2 MIT@ZIF‐8 Nanostructures Release Mitochondria in Response to Acidic pH Environment

**FIGURE 2:**
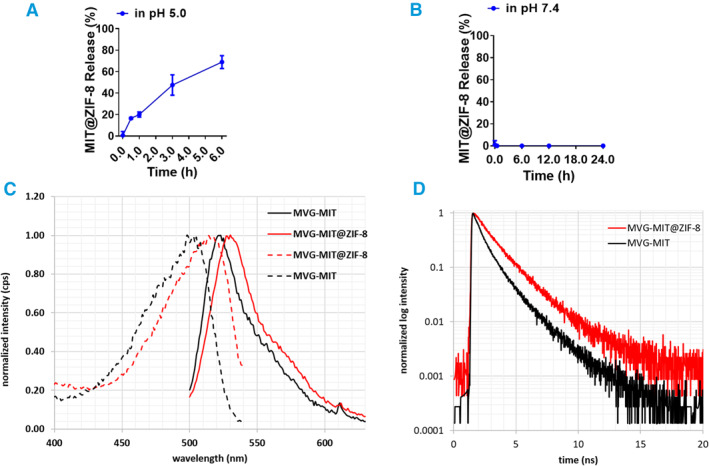
Dynamic analysis of MIT@ZIF‐8 nanostructures. (A) pH‐responsive release of mitochondria from MIT@ZIF‐8 in a pH 5.0 HAc buffer. (B) pH‐responsive release in a pH 7.4 saline solution. (C, D) Measurement of the average fluorescence lifetime in MIT@ZIF‐8. (C) Fluorescence and excitation spectra for MVG‐labeled mitochondria in both the presence and absence of ZIF‐8. (D) Fluorescence decay curves for MVG‐labeled mitochondria in the presence and absence of ZIF‐8. The excitation wavelength (*λ*_ex_) was set at 483 nm, and the decay was monitored at 520 nm. A cutoff filter was employed with the detector to block the excitation, preventing disturbances from scattering.

Given that ZIF‐8 is a pH‐responsive MOF, we investigated whether our synthesized MIT@ZIF‐8 NPs could be released in a pH‐dependent manner. Following the radio immunoprecipitation assay (RIPA) lysis of the released mitochondrial after centrifugation, we utilized a BCA protein assay kit to analyze protein concentration, with a detection limit of 25 μg/mL. The normalization analysis was based on the total protein content of the encapsulated mitochondria of the same volume (Supporting Information S1: Figures S7, S8). The results demonstrated that approximately 70% of the mitochondria in MIT@ZIF‐8 NPs were released after 6 h at pH 5.0, compared to pH 7.4 (Figure 2A,B).

To further assess whether mitochondria were fully encapsulated by ZIF‐8, we conducted fluorescence measurements using the mitochondrial‐binding fluorescence dye MVG. We recorded steady‐state fluorescence and excitation spectra, along with fluorescence decay curves, for MVG‐labeled mitochondria both with and without ZIF‐8 coating. Both the excitation and fluorescence spectra experienced a 10 nm shift toward the red (Figure 2C) and the fluorescence decay duration was noticeably longer in the presence of ZIF‐8 (Figure 2D). The decay curves were fitted with two‐exponential equation. While both lifetimes increased somewhat (Supporting Information S1: Table S1), the most significant change occurred in the amplitude of the longer‐lived component, which rose from 29% to 55%. Consequently, the average fluorescence lifetime <*τ*> increased from 0.6 ns without ZIF‐8 to 1.1 ns with ZIF‐8 present. These changes indicate the alterations in the environment of the MVG probe due to the encapsulation of MIT with ZIF‐8.

Together, our fluorescence lifetime measurements combined with cut section analysis of MIT@ZIF‐8 NPs, demonstrate that the mitochondria are fully encapsulated by ZIF‐8. Furthermore, they can be released in a pH‐dependent manner.

### 3.3 The Protective Effects of ZIF‐8 Coating on Mitochondria Activity

Since ZIF‐8 coating can extend the fluorescence lifetime of the fluorescent dye MVG when bound to the mitochondrial surface under laser excitation, we explored whether ZIF‐8 coating could also maintain the mitochondrial activity, specifically focusing on mitochondrial membrane potential (ΔΨm) and ATP synthesis capability. The ΔΨm generated by proton pumps (Complexes I, III and IV), is crucial for energy storage during oxidative phosphorylation. Along with the proton gradient (ΔpH), ΔΨm contributes to the transmembrane potential of hydrogen ions, which is utilized to produce ATP. We initially assessed the staining pattern of ΔΨm‐dependent tetramethylrhodamine, methyl ester (TMRM) dye (red) and ΔΨm‐independent MVG dye (green) in living cells under varying conditions (Supporting Information S1: Figures S9, S10). Our findings indicated that TMRM staining effectively reflects ΔΨm, whereas MVG staining accurately measures mitochondria biomass.

**FIGURE 3:**
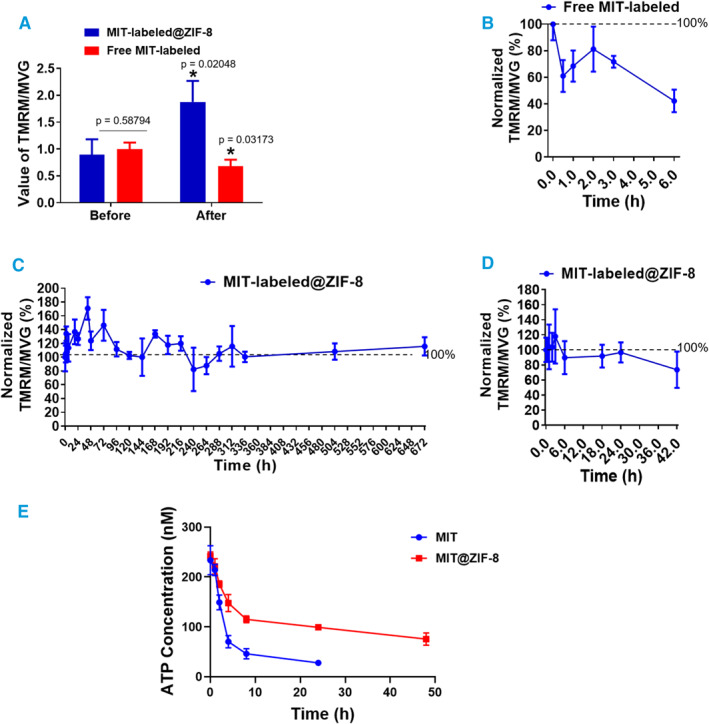
ZIF‐8 coating has a protective effect on the mitochondrial activity. Mitochondria, labeled with MVG and TMRM, were isolated from MCF‐10A cells. The relative membrane potential of these mitochondria is determined by normalizing the TMRM/MVG ratio using absorbance values following fluorescence excitation. (A) The TMRM/MVG ratios of mitochondria are depicted for each group, both before and after MOF formation. An asterisk (*) indicates comparisons with an equivalent number of cells‐deriving free MIT‐MVG/TMRM prior to MOF formation. (B) The dynamic curves show the relative membrane potential of free mitochondria in a mitochondria‐specific buffer kept on ice. (C) The dynamic curves illustrate the relative membrane potential of ZIF‐8‐coated mitochondria maintained in 0.9% NaCl at RT for up to 4 weeks. (D) The dynamic curves display the relative membrane potential of ZIF‐8‐coated mitochondria in a cell lysis solution (RIPA) at RT for 48 h. (E) An ATP synthesis capability assay comparing ZIF‐8‐coated mitochondria (MIT@ZIF‐8) with uncoated mitochondria (MIT).

To account for potential variations in the number of mitochondria encapsulated by ZIF‐8 at different measurement intervals, we normalized the ΔΨm value by the relative mitochondrial biomass, determined by the TMRM/MVG ratio after fluorescence excitation at 490 and 548 nm, respectively. We found that after ZIF‐8 mineralization, the relative membrane potential of mitochondria increases, whereas that of the non‐mineralized mitochondria decrease (Figure 3A). Remarkably, the mitochondrial membrane potential of non‐mineralized mitochondria decreases significantly to 40% within the first 6 h even when preserved on ice in the mitochondria isolation buffer (Figure 3B). In contrast, ZIF‐8‐mineralized mitochondria (MIT@ZIF‐8) maintained their relative membrane potential in 0.9% NaCl at RT, for up to 4 weeks (Figure 3C). Moreover, we investigated whether the ZIF‐8‐coating layer offers protective effects on mitochondria under extreme conditions. In this experiment, we immersed MIT@ZIF‐8 in typical cell lysis solution—RIPA at RT and found that their relative ΔΨm remained stable for at least 24 h (Figure 3D).

We then performed the ATP synthesis capability assay to compare ZIF‐8‐coated mitochondria (MIT@ZIF‐8) with uncoated mitochondria (MIT). Our results showed that isolated free mitochondria retained their ATP production capacity before encapsulation, whereas the mitochondria in the uncoated group (MIT) could not sustain this capacity over time. In contrast, the mitochondria in the coated group (MIT@ZIF‐8) successfully preserved ATP production even after 48 h (Figure 3E, Supporting Information S1: Figure S11).

Together, these results suggest that ZIF‐8 coating exerts protective effects on the activity of isolated mitochondria.

### 3.4 Modification of MIT@ZIF‐8 NPs by PEI and Tat

**FIGURE 4:**
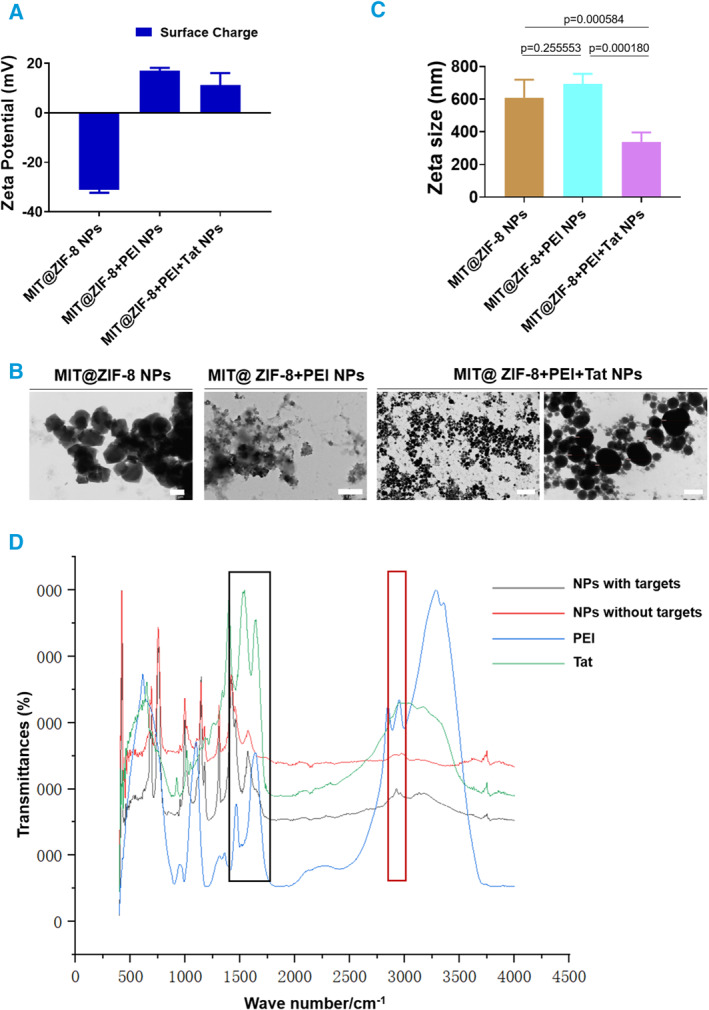
Comparison analysis of MIT@ZIF‐8 NPs and their modified counterparts with PEI and Tat. (A) The Zeta potential analysis of unmodified MIT@ZIF‐8 NPs and those modified by PEI and both PEI + Tat. (B) TEM analysis of MIT@ZIF‐8 NPs, MIT@ZIF‐8+PEI, and MIT@ZIF‐8+PEI + Tat. Scale bars, 1 μm except for the magnified image of MIT@ZIF8+PEI + Tat on the right (200 nm). (C) A Zeta size analysis compares the size variations among MIT@ZIF‐8 NPs, MIT@ZIF‐8+PEI NPs, and MIT@ZIF‐8+PEI + Tat NPs. (D) FT‐IR analysis of the infrared spectra of PEI and Tat powders, and the NPs powders from both groups (NPs with targets: modified MIT@ZIF‐8 NPs by PEI and Tat; NPs without targets: unmodified MIT@ZIF‐8 NPs). The two rectangular boxes indicate the surface characteristic peak positions, with the black box representing NPs surfaces modified with Tat, and the red box indicating those modified with PEI. PEI, polyethyleneimine; Tat, cell‐penetrating peptide Tat; NPs, nanoparticles; FT‐IR, fourier transform infrared spectrometer.

The typical size of mitochondria ranges from 500 to 1000 nm, which poses challenges for cellular entry. To address this, we incorporated cationic polymer polyethylenimine (PEI), a widely used gene vector and transfection reagent for the transient transfection and expression of recombinant proteins or virus vectors [34], into the synthesis process of MIT@ZIF‐8 NPs. This modification was intended to change the surface charge to positive (Figure 4A), However, TEM observation revealed that the aqueous dispersion of PEI‐modified MIT@ZIF‐8 NPs was poor (Figure 4B). To further enhance the permeability of MIT@ZIF‐8 NPs, we integrated the cell‐penetrating peptide Tat [35], into the synthesis process. Notably, the aqueous dispersion of MIT@ZIF‐8 NPs concurrently modified by PEI and Tat showed improvement (Figure 4B). Moreover, the particle size measurements showed that the average size of MIT@ZIF‐8 NPs decreased from 608.4 to 337.5 nm following the dual modification with PEI and Tat (Figure 4C).

To confirm the successful surface functionalization, we performed Fourier Transform Infrared Spectrometer (FT‐IR) analysis (Figure 4D). The infrared spectra showed clear distinctions between the modified and unmodified NPs. A characteristic Tat peak appeared around 1500 cm^−1^ (highlighted in a black box), while a PEI peak was observed near 3000 cm^−1^ (highlighted in a red box). These results validated the surface functionalization of the MIT@ZIF‐8 NPs with Tat and PEI.

These results suggested that incorporating the cell‐penetration peptide Tat and the cationic polymer PEI into the ZIF‐8 layer enhances the MIT@ZIF‐8 NPs. This optimization affects characteristics such as size, surface charge, and dispersion in aqueous environments, potentially improving the delivery efficiency into recipient cells.

### 3.5 Analysis of Cellular Uptake and Behavior After the Delivery of Non‐tumorigenic Mitochondria‐Derived MIT@ZIF‐8

**FIGURE 5:**
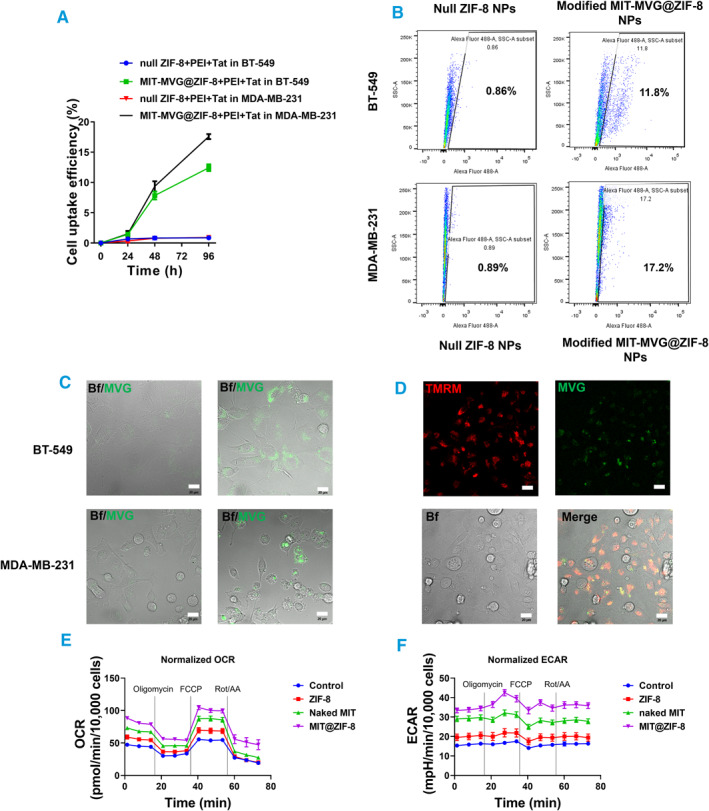
Analysis of cellular uptake in breast cancer cells using modified MIT‐MVG@ZIF‐8 derived from healthy breast cells. (A, B) Dynamic curves of cell uptake efficiency: MVG‐labeled MIT@ZIF‐8 uptake was assessed at indicated time points in breast cancer cells through flow cytometry analysis. (A) Displays the dynamic curves of cell uptake efficiency. (B) Represents a typical flow cytometry result showing cell uptake efficiency on day 4 post‐incubation with MVG‐labeled MIT@ZIF‐8 in breast cancer cells. (C) CLSM analysis of cell uptake on day 2 following incubation of modified MIT‐MVG@ZIF‐8 NPs with live cells. Scale bars, 20 μm. (D) CLSM analysis of endogenous and exogenous mitochondria in TMRM‐stained live MDA‐MB‐231 cells on day 2 after the uptake of modified MIT‐MVG@ZIF‐8 NPs. Scale bars, 20 μm. Bf, Brightfield. (E, F) Seahorse assay in MDA‐MB‐231 cells. (E) Shows normalized value of oxygen consumption rate (OCR), and (F) shows normalized value of extracellular acidification rate (ECAR) for each group.

Finally, as an illustrative therapy example, we provided proof‐of‐concept data showcasing the feasibility and biological effects of delivering modified non‐tumorigenic MIT@ZIF‐8 into cancer cells. We prepared MVG‐labeled MIT@ZIF‐8 from non‐tumorigenic mammary epithelial cells (MCF‐10A), modified by PEI and Tat (modified MIT‐MVG@ZIF‐8). Through flow cytometry analysis, we plotted the kinetic curve of the modified MIT‐MVG@ZIF‐8 entry into recipient breast cancer cells (BT‐549 and MDA‐MB‐231) (Figure 5A). At day 4 following incubation, the cell uptake efficiencies were 11.8% and 17.2% for BT‐549 and MDA‐MB‐231 cells, respectively (Figure 5A, B). Under CLSM, modified MIT‐MVG@ZIF‐8 (green) can be clearly observed within the recipient breast cancer cells (Figure 5C). To assess whether the delivered exogenous mitochondria fused with the mitochondria of the recipient cells, we performed live cell TMRM staining for mitochondria within the recipient cells (red) and subsequently introduced the modified MIT‐MVG@ZIF‐8 (green). The results showed occurrences of fusion between transplanted MIT (green) and endogenous mitochondria (red), resulting in visual yellow fusion (Figure 5D). To examine whether mitochondrial function in recipient cells was enhanced by our delivery method, we performed a seahorse assay on the MDA‐MB‐231 cells receiving the treatments from each group. We observed an increase in both the oxygen consumption rate (OCR) (Figure 5E) and the extracellular acidification rate (ECAR) (Figure 5F) in the MIT@ZIF‐8‐treated group, compared to other groups, indicating an improvement in mitochondrial function in recipient cells after mitochondrial delivery.

**FIGURE 6:**
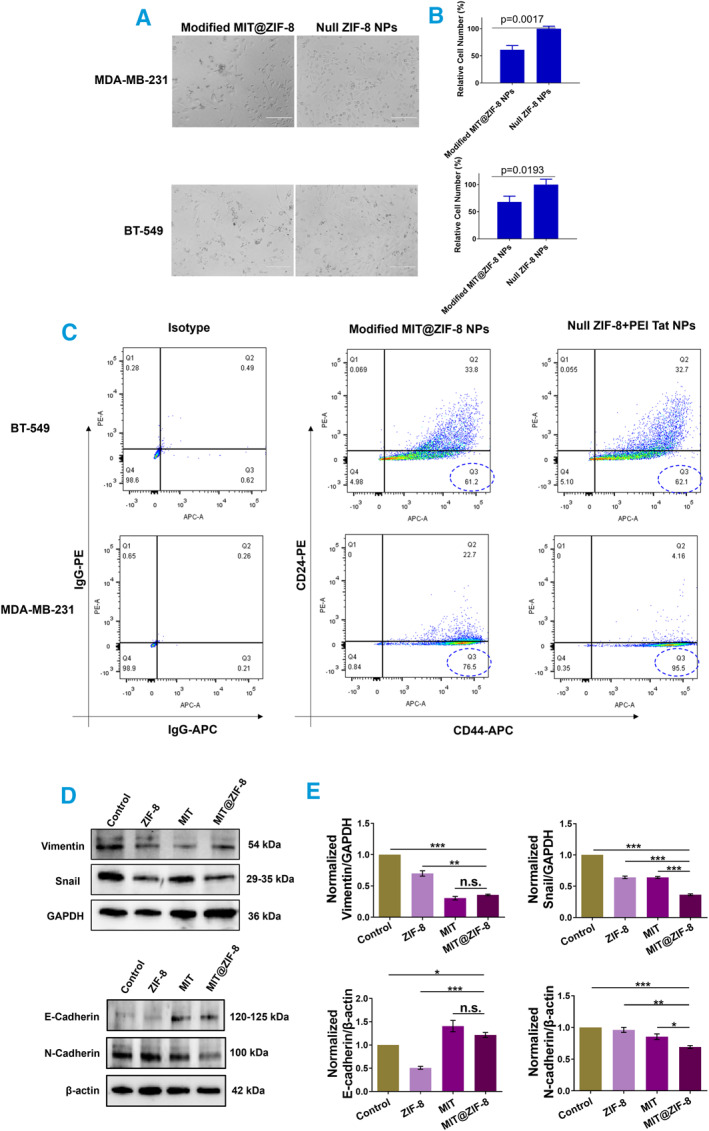
Analysis of cell behavior following the transfer of modified mineralized healthy mitochondria (MIT@ZIF8) into cancer cells. (A) Brightfield images showing cancer cells before and after delivery of modified MIT@ZIF‐8. Scale bars, 200 μm. (B) Comparative analysis of cell numbers between the two groups. (C) Assessment of breast cancer stem cell population (CD44^+^/CD24^‐^) analyzed by flow cytometry, conducted after the delivery of modified MIT@ZIF‐8 into two cell lines. Isotype IgG‐APC and isotype IgG‐PE were added to each cell line as controls. (D, E) Western blot analysis of expression changes of epithelial‐mesenchymal transition marker proteins in each group of MDA‐MB‐231 cells, with values normalized to the control group, set as 1. n.s., not significant. *p < 0.05, **p < 0.01, ***p < 0.001.

Next, we evaluated the biological effects of delivering modified MIT@ZIF‐8 NPs from non‐tumorigenic breast cells (MCF‐10A) mitochondria to breast cancer cells, focusing on cell proliferation and cancer stem cell population. Our results showed a significant inhibition in the proliferation of both breast cancer cell lines following delivery of MCF‐10A mitochondria‐derived modified MIT@ZIF‐8 NPs (*p* = 0.0017, *p* = 0.0193, respectively) (Figure 6A,B). Flow cytometry analysis revealed a decrease in the breast cancer stem cell population (CD44^+^CD24^‐^) in MDA‐MB‐231 cells from 95.5% to 76.5%, while in BT‐549 cells, the percentage remained nearly constant (62.1%–61.2%) (Figure 6C). Our finding suggests that dysfunctional mitochondria in MDA‐MB‐231 cells contribute to maintaining stemness, which can be inhibited by transferring mitochondria NPs from non‐tumorigenic cells. In contrast, BT‐549 cells, seem to be influenced by other major stemness drivers, or the transplantation efficiency might be lower for BT‐549 cells (Figure 5B). In addition, we examined the expression levels of epithelial‐mesenchymal transition (EMT) markers including E‐cadherin, N‐cadherin, vimentin, and Snail in MDA‐MB‐231 cells, as these markers are known to be responsible for cancer stem cell properties [36]. Both MIT@ZIF‐8 NPs and isolated mitochondria (MIT) treatments led to decreased expression levels of mesenchymal markers N‐cadherin, Vimentin and Snail, while increasing the expression of the epithelial markers E‐cadherin compared to control groups or ZIF‐8 null NP groups. These results indicate that MIT@ZIF‐8 NP transplantation can inhibit EMT in breast cancer cells.

Taken together, the cell uptake efficiency of the modified MIT@ZIF‐8 NPs was 12%–17% in BT‐549 and MDA‐MB‐231 cells. Some of the transplanted MITs integrated with the endogenous mitochondria. A notable decrease in cell growth, the CD44^+^CD24^‐^ population, and the EMT status was observed in the recipient cancer cells.

## 4 Discussion

Mitochondrial transplantation holds significant promise for addressing cancer and other metabolic diseases. However, challenges such as the swift decline in mitochondrial function and low transplantation rates hinder its effectiveness. To address these issues, we have developed a highly efficient aqueous synthesis technique that facilitates the rapid creation of ZIF‐8‐coated mitochondrial nanocomposites through a one‐pot synthesis method. These nanocomposites were meticulously characterized using TEM, CLSM, Zetasizer Nano ZS and Time‐Correlated Single‐Photon Counting (TCSPC). We assessed changes in mitochondrial membrane potential and ATP synthesis capability and introduced PEI and Tat for the surface modification of MIT@ZIF‐8. This optimization enhances delivery efficiency into cancer cells, allowing us to better evaluate their biological effects.

For a considerable period, freshly isolated mitochondria have been the sole option for biochemical and cellular experiments, mainly due to the critical challenge of maintaining mitochondrial activity in vitro over time. In 2006, Nukala et al. demonstrated for the first time that isolated rat brain mitochondria could be successfully cryopreserved in 10% dimethyl sulfoxide (DMSO) and later restored for use, akin to cell cryopreservation [37]. Here, we present a novel approach inspired by natural biomineralization, demonstrating for the first time that isolated healthy mitochondria can be effectively coated and protected with MOF nanosystems, such as ZIF‐8, and then delivered into cancer cells as a therapeutic agent. This pioneering technique ensures the preservation of membrane potential at RT, facilitating their use in subsequent applications. Our research extends the protective effects of ZIF‐8 on macromolecules including proteins, antibodies and bioactive enzymes [24, 38, 39], as well as yeast cells [40, 41] into the realm of cellular organelle mitochondria.

Our findings demonstrate that ZIF‐8‐coated mitochondria show improved preservation and resilience under extreme conditions. However, under current conditions, this coating presents a challenge for further examination of the functionality of the encapsulated mitochondria. To analyze mitochondrial function, it is necessary to release them from the nanocomplexes in a pH‐responsive manner under acidic conditions. Consequently, we opted to measure the mitochondrial membrane potential in situ, consistent with Wang et al. reported [42], as it is a crucial indicator of mitochondrial activity and can be detected using fluorescent dyes. Our results indicated that the membrane potential of ZIF‐8‐mineralized mitochondria (MIT@ZIF‐8) is sustainable in 0.9% NaCl at RT for up to 4 weeks. This suggests that measurement of relative mitochondrial membrane potential in situ within MIT@ZIF‐8 NPs solutions, normalized by the ratio of two different mitochondrial dyes, TMRM and MVG, after fluorescence excitation, is a straightforward, non‐invasive, and reliable method.

A study conducted in 2019 demonstrated that mitochondrial transfer can occur under human pathological conditions. Specifically, when the body is infected, reactive oxygen species (ROS)‐mediated PI3K activation prompts the transfer of mitochondria from stromal cells to hematopoietic stem cells [15]. Researchers have also started directly delivering freshly isolated mitochondria into target cells for endocytosis, a process known as “mitochondrial transplantation”, which has shown some therapeutic benefits [16, 17, 19]. For example, Elliott et al. [43] isolated and purified mitochondria from non‐tumorigenic MCF‐12A breast epithelial cells and co‐cultured them with breast cancer cells. They found that mitochondria from non‐tumorigenic cells can spontaneously enter multi‐drug‐resistant breast cancer cells, slowing ATP production by antagonizing the glycolysis pathway of tumor cells. This disruption causes the energy supply rate and the rate of malignant proliferation to fall out of synchronization, significantly inhibiting the proliferation and spread of tumor cells. However, these studies must exclusively use freshly isolated mitochondria each time, as they are highly sensitive to processing time and conditions, leading to considerable variation between batches. Thus, it often results in large variation between batches. Additionally, managing bare mitochondria for real‐world clinical use poses challenges, since they cannot be stored for extended periods.

The inherent ability of bare mitochondria to penetrate biological barriers is limited [44], as they typically range in size from 500 to 1000 nm. Like NPs in this range, they do not easily enter cells. It's reported that the size of NPs significantly affects their circulation duration, targeting abilities, and cellular uptake [45]. Therefore, we hypothesized that mitochondria coated with ZIF‐8 could be modified during the one‐pot synthesis process to improve their properties. Given that PEI is a common transfection reagent and Tat is a widely used cell‐penetrating peptide, we experimented with modifying ZIF‐8‐coated mitochondria by integrating PEI and Tat to improve cellular uptake efficiency. Interestingly, we observed that the size of the modified MIT@ZIF‐8 NPs with PEI + Tat decreased from an average of around 600 nm to around 300 nm, alongside an improvement in their aqueous dispersion.

Cell‐penetrating peptides possess the remarkable capability to transport large quantities of proteins, nucleic acids, NPs, and other substances into target cells [35]. They facilitate this process through both ATP‐dependent endocytosis and ATP‐independent membrane translocation pathways [46]. We concluded that the cell‐penetrating peptide Tat not only enhances the entry of MIT@ZIF‐8 NPs into cells, but might also function similarly to the reported “protein crown (PC)” by mediating the interactions between proteins and NPs [47, 48]. This capability may help prevent NP aggregation and mitigate NP toxicity [49, 50]. Furthermore, the modification of NPs with PEI alters their surface charge from negative to positive, potentially enhancing their retention of NPs on the cell membranes via electrostatic adsorption [45]. These findings underscore the significance of combining PEI and Tat functionalization for effective intracellular transplantation of ZIF‐8‐coated mitochondria. Our technique offers an alternative to traditional methods that involve introducing sequences into mitochondria to increase cell‐specific targeting, achieving desired functionality through non‐invasive modifications to the MOF layers without resorting to genetic manipulation of the coated mitochondria.

Recent evidence indicates that mitochondria‐rich extracellular vesicles (EVs) from donor cells can integrate into the mitochondrial network of recipient cells, thereby influencing their biological functions [51, 52, 53, 54]. Meanwhile, ZIF‐8 material is known for its excellent biocompatibility, and structural stability under physiological conditions. It enters the lysosome through an ATP‐dependent endocytosis pathway. Under acidic conditions, the imidazole ring, a degradation product of ZIF‐8, facilitates the escape of cargo—such as the transferred mitochondria in this study—from the endosomes [55, 56]. Consistently, our observations demonstrated the fusion of exogenously delivered ZIF‐8‐coated mitochondria with endogenous mitochondria. Moreover, in recipient breast cancer cells, a significant reduction in cell growth, EMT status, and the cancer stem cell‐enriched CD44^+^CD24^‐^ population as observed post‐delivery. These indicated that mitochondrial delivery substantially decreased stemness in breast cancer cells. Specifically, in our study, the CD44^+^CD24^‐^ population significantly decreased in MDA‐MB‐231 cells, while it remained virtually unchanged in BT‐549 cells after mitochondrial delivery. This implies that the anti‐cancer effect of delivered normal mitochondria may depend on the recipient cells' characteristics or the efficiency of the transplantation. In 2023, Watson et al. reported that in glioblastoma, cancer cells acquire mitochondria from astrocyte cells. This mitochondrial transfer alters metabolism, signal transduction, and epigenome remodeling, promoting cancer cells' stem cell‐like properties, such as self‐renewal and tumorigenicity [57]. This finding contradicts earlier reports [16, 17, 19, 43]. It also suggests that the effectiveness of mitochondrial delivery for tumor treatment may be context‐dependent, influenced by several factors. These include the condition of the delivered mitochondria (e.g., mitochondria components, damaged mitochondria [58] or bioactive mitochondria), the study models used (e.g., in vivo observation of mitochondrial transfer behavior [15] or in vitro manipulation of isolated and purified mitochondria for cell delivery) [16, 17, 19, 43], the type or subtype of cancer cells, and even the process of mitochondrial exocytosis in cancer cells themselves [53]. Further investigation is necessary to gather more evidence in these areas.

To investigate the variations in potential biological mechanisms across diverse models, the mitochondria transfer technology, developed through MOF biomineralization in this study, offers a promising research tool.

## 5 Conclusion

**FIGURE 7:**
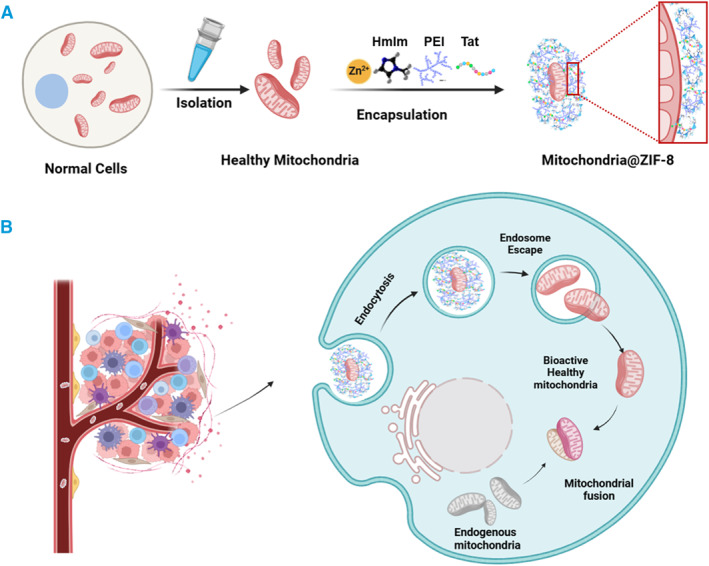
Schematic figure for illustrating the process of biomineralizing mitochondria within MOFs to promote mitochondria transplantation from non‐tumorigenic cells into cancer cells. (A) Schematic illustration of the isolation of healthy mitochondria and their encapsulation within MOFs (MIT@ZIF‐8), using a one‐pot synthesis method. This involves the rapid mixing and reaction of isolated mitochondria with Zn^2+^ ions and HmIm at RT. To optimize MIT@ZIF‐8, a cell‐penetrating peptide, Tat, and the cationic polymer PEI are introduced into the ZIF‐8 layer, thereby increasing delivery efficiency to recipient cells. (B) The diagram further details the intracellular delivery, and endosomal escape of MIT@ZIF‐8, followed by the release of exogenous mitochondria within the cells. These mitochondria then dynamically fuse with endogenous mitochondria, completing the transplantation process. HmIm, 2‐methylimidazole; PEI, polyethyleneimine; Tat, cell‐penetrating peptide Tat.

Taken together, our study broadens the application of MOFs, especially ZIF‐8 MOFs, within the biomedicine field. Our findings demonstrate that ZIF‐8‐coated mitochondria not only encapsulate and protect biological macromolecules such as DNA, enzymes, and proteins, but also exhibit stability at RT, resistance to harsh external conditions and enhanced ability for cellular uptake and functional activity when modified with PEI and Tat (Figure 7). These modifications allow the mitochondria to enter recipient cells and exert biological effects more efficiently. While our study does not focus on specific mechanisms, it conceptually validates the feasibility of manually manipulating mitochondrial delivery. It also offers new strategies and insights for advancing the large‐scale biomedical use of mitochondria in treating human diseases in the future.

## 6 Experimental Section/Methods

### 6.1 Cell Culture

Human breast cancer MDA‐MB‐231 cells and BT‐549 cells, and human non‐tumorigenic breast cells MCF‐10A were cultured as previously reported [59].

### 6.2 ZIF‐8 Encapsulation for Freshly Isolated Mitochondria

One day prior to isolation, mitochondria in live cells were stained with a final concentration of 100–200 nM MitoView Green at 37°C protected from light, according to the manufacture's protocol (MVG, Biotium, San Francisco Bay Area, the USA). To verify proper staining, a test was performed under a fluorescent microscope. Approximately 10 dishes of MCF‐10A cells, reaching 80%–90% confluence, were digested for 20 min at 37°C in a cell incubator prior to cell counting. Mitochondria were freshly isolated using the mitochondria isolation kit for cultured cells according to the manufacture's protocol (ThermoFisher Scientific, 89874, Waltham, Massachusetts, USA). All reagents were kept in an ice bath before and during isolation. Mitochondria pellets were then added to a 2‐methylimidazole solution at 160 mM, followed by dropping zinc acetate solutions (40 mM) into the 2‐methylimidazole solution containing mitochondria with stirring for 2–3 min, and allowed to settle for at least 10 min in RT. For the modification of MIT@ZIF‐8 NPs by PEI and Tat, 1–2 μg/mL (final concentration) of branched PEI (average Mw ∼25,000, Sigma‐Aldrich) and 0.5–1 mg/mL (final concentration) of the cell‐penetrating peptide Tat (sequence: GRKKRRQRRR, Synpeptide Co., Ltd., Shanghai, China), were added during the process of NPs synthesis. The mixture was centrifuged at 13,000 rpm for 5 min, with the supernatant subsequently discarded. The MIT@ZIF‐8 NPs were then washed twice with a 0.9% NaCl solution. For biological testing, NPs were added to cell culture medium at a 20‐fold–50‐fold dilution for at least 48 h.

### 6.3 Characterization of MIT@ZIF‐8 NPs

To examine the morphology of the synthetic NPs, grids for the TEM samples were briefly immersed in the complex solution for each setup, then removed and placed on absorbent paper to dry overnight. For assessing the mitochondria encapsulated within the NPs, the pellets of the MIT@ZIF‐8 were collected and embedded in resin to prepare sections for further TEM analysis. The TEM samples were examined with a JEOL 1400 Plus (USA). Particle size was measured through dynamic light scattering with a Zetasizer Nano ZS (Malvern Instruments Ltd., UK). The NPs surface zeta potential was measured with a Zetasizer Nano ZS by using disposable folded capillary cells (DTS1070, Malvern, UK).

### 6.4 Confocal Laser Scanning Microscopy Analysis

For the NPs, solution of the labeled NPs was diluted 50 to 100 times with physiological saline. This diluted solution was then carefully added dropwise onto a glass slide and gently covered with a cover slip to prevent bubble formation. To observe endogenous mitochondria and the fusion between endogenous mitochondria and phagocytic ZIF‐8‐coated mitochondria within cells, pre‐treated endogenous and exogenous mitochondria were subjected to CLSM observation in living cells. All samples were analyzed using confocal laser scanning microcopy (CLSM) with the Zeiss LSM880 system (Oberkochen, Germany).

### 6.5 The Encapsulation Efficiency and Release Efficiency

The labeled NPs were photographed by CLSM as described above. To determine the mitochondrial encapsulation efficiency (%), the proportion of bicolor labeled NPs (i.e., MIT‐MVG@ZIF‐8‐Cy5) among all kinds of NPs (MIT‐MVG@ZIF‐8‐Cy5 and null@ZIF‐8‐Cy5) was calculated relative to the total NPs, which included MIT‐MVG@ZIF‐8‐Cy5 and null@ZIF‐8‐Cy5. For the release of mitochondria from the NPs, a sodium acetate buffer at pH 5.0 and a 0.9% physiological saline solution at pH 7.0 were used, respectively. After RIPA lysis of the released mitochondria collected by high speed centrifugation, the protein concentration was determined using a BCA protein assay (Pierce BCA Protein Assay Kit, ThermoFisher Scientific). The normalization analysis was conducted based on the total protein content of the encapsulated mitochondria within an equivalent volume.

### 6.6 Measurement of the Relative Membrane Potential of the Mitochondria

One day prior to isolation, we performed double staining of mitochondria in live cells with a final concentration of 100 nM MitoView Green (MVG, Biotium, San Francisco Bay Area, USA) and Image‐iT TMRM Reagent (ThermoFisher Scientific, I34361, Waltham, Massachusetts, USA) at 37°C protected from light, according to the manufacture's protocol. A part of the stained mitochondria underwent mineralization, while the non‐mineralized mitochondria served as controls. The fluorescence signal values were read with a Varioskan LUX multimode microplate reader (ThermoFisher Scientific, Waltham, Massachusetts, USA). The relative membrane potential of the ZIF‐8‐coated mitochondria was calculated as the ratio of the TMRM fluorescence signal to the MVG fluorescence signal, following fluorescence excitation at 490 and 548 nm, respectively.

### 6.7 ATP Synthesis Capability Assay of the Mitochondria

The ATP concentration was assessed using an ATP assay kit from ThermoFisher Scientific (Waltham, Massachusetts, USA). A standard curve ranging from 0 to 1000 nM was created by diluting an ATP standard. Mitochondrial protein levels were measured via a BCA protein assay (Pierce BCA Protein Assay Kit, ThermoFisher Scientific), following mitochondria isolation and encapsulation procedures. Both experimental and standard samples were placed into 96‐well plate. The reaction solution was added to achieve a final volume of 100 μL per well, and all samples were analyzed. After a 15‐min incubation at room temperature, luminescence was measured using a Varioskan FLASH (ThermoFisher Scientific, Waltham, Massachusetts, USA). ATP concentrations were then calculated based on the standard curve.

### 6.8 Fluorescence Measurements

Steady‐state fluorescence and excitation spectra were recorded with an FLS‐1000 spectrofluorometer (Edinburgh Instruments, UK). Time‐resolved fluorescence was measured with a time‐correlated single‐photon counting (TCSPC) system (Pico‐Quant GmBH, Chaussee, Germany) consisting of a PicoHarp 300 controller and a PDL 800‐B driver. Freshly encapsulated samples labeled with MVG and unlabeled mitochondria were excited with the pulsed diode laser head LDH‐P‐C‐485 at 483 nm, with a time resolution of 64 ps. Signals were captured using a microchannel plate photomultiplier tube (Hamamatsu R2809U). The influence of scattered excitation light was minimized by placing a cutoff filter (transmission > 500 nm) in front of the monitoring monochromator. Fluorescence decays were collected at 520 nm. The instrumental response function (IRF) was measured separately. The decay data were deconvoluted and fitted by using an iterative least‐squares method applied to the sum of two exponents:

$$I\left(t\right)=\sum_{i}a_{i}e^{-t/\tau_{i}}$$

In this equation, *τ*_*i*_ is the fluorescence lifetime and *a*_*i*_ is the amplitude (pre‐exponential factor) of each decay component. The mean amplitude weighted lifetime 〈τ〉 was calculated using equation:

$$<\tau \geq \frac{\sum a_{i}\tau_{i}}{\sum a_{i}}$$

### 6.9 FT‐IR Analysis

Mitochondria were isolated following the experimental procedure. One group underwent modification with Tat and PEI during ZIF‐8 mineralization, whereas the control group did not receive any targeting agents. Subsequently, both groups were freeze‐dried. After freeze‐drying, a FT‐IR Spectrometer (PerkinElmer, Waltham, Massachusetts, USA) was used to analyze and compare the infrared spectra of PEI, Tat powders, and the NPs powders from both groups.

### 6.10 Seahorse Assay

Mitochondrial OCR and ECAR were measured using the Seahorse XFe 96 Analyzer (Agilent Technologies, California, USA) in conjunction with the Cell Mito Stress Test Kit. Briefly, MDA‐MB‐231 cells were collected and resuspended at a density of 2 × 10^4^ cells. then a 1.2 mL aliquot of this suspension was transferred into an Eppendorf tube. For the ZIF‐8, MIT, and MIT@ZIF‐8 groups, 10 μL of the respective treatment was introduced, while an equivalent volume of medium was added to the Blank group. Subsequently, 180 μL of the cell suspension was dispensed into the corresponding cartridge wells, with 12 parallel wells for each group. The plates were then returned to the incubator for further cultivation. After 48 h, measurements were performed, following the kit's instructions. The reagents were used at final concentrations of 1 μM oligomycin (Oligo), 0.75 μM carbonyl cyanide‐4‐(trifluoromethoxy) phenylhydrazone (FCCP), and 0.5 μM rotenone/antimycin A (Rot/AA). To accurately normalize rates to cell counts, Hoechst 33342 was added to the Rot/AA solution at a final concentration of 20 μM to stain cell nuclei. After the assay, the cell plate was transferred to the Cytation 3 Cell Imaging Reader (BioTek, Winooski, VT, USA) for imaging and analysis with Gen5 software (BioTek), enabling cell count determination for each well.

### 6.11 Western Blot Analysis

Proteins separated by SDS‐PAGE gel electrophoresis were transferred onto a PVDF membrane (Immobilon FL 0.45 μm, Merck, Rahway, USA) using the semi‐dry transfer method in the Transblot SD apparatus (Bio‐Rad). For proteins with a molecular weight exceeding 60 kDa, the transfer was performed at 15 V for 1 h, while for those with a molecular weight below 60 kDa, the transfer was performed at 15 V for 40 min. After the transfer, the membrane was blocked with 5% BSA for 1 h, followed by overnight incubation with the primary antibody. The membrane was then washed, incubated with the secondary antibody, and washed again. Detection was achieved using the ChemiDoc Imaging System (Bio‐Rad, California, USA). Signals detected by the scanner and band intensities were quantified using Image J software. Detailed information on antibodies is provided in Supporting Information S1: Table S2.

### 6.12 Flow Cytometry Analysis

For cellular uptake analysis, the percentage of cells that internalize MVG‐labeled MIT@ZIF‐8 NPs was analyzed using the FITC channel. For analysis of CD44 and CD24 expression levels in the cells, flow cytometry samples were prepared according to the manufacturer's protocol (Invitrogen, Waltham, Massachusetts, USA) as previously reported [59]. All samples underwent analysis used with a BD LSR Fortessa analyzer according to BD LSRFortessa instrument instruction (16.05.2019 edition) by Cell Imaging and Cytometry, Turku Bioscience, Finland (https://bioscience.fi/services/cell‐imaging/services/). Detailed information of antibodies and isotype IgG is provided in Supporting Information S1: Table S2.

### 6.13 Statistical Analyses

Data were expressed as means with standard deviations (SD), with at least three replicates for each group. Statistical graphs were generated using GraphPad Prism 8 (GraphPad Software Inc., San Diego, CA, USA) or Origin 6.1 (OriginLab, Northampton, MA, USA). The statistical significance of differences between the two groups was determined by unpaired two‐tailed t‐tests. A *p*‐value of less than 0.05 was considered statistically significant, while a *p*‐value greater than 0.05 was regarded as not significant (ns).

## Author Contributions

Conceptualization, J.‐N.Z., C.L., and H.Z.; Methodology, J.‐N.Z., C.L., Y.W., Y.G., X.‐Y.X., E.V.‐L, O.K., and J.Y.; Validation, J.‐N.Z., C.L., and Y.W.; Formal Analysis, J.‐N.Z., C.L., Y.W., and H.Z.; Investigation, J.‐N.Z., C.L., Y.W., Y.G., J.Y., and H.Z.; Data Curation, J.‐N.Z., C.L., Y.W., and E.V.‐L; Writing—Original Draft Preparation, J.‐N.Z., C.L., and Y.W.; Writing—Review & Editing, J.‐N.Z., C.L., J.Y, A.M.F., and H.Z.; Visualization, J.‐N.Z., C.L., Y.W., and E.V.‐L; Funding Acquisition, H.Z., J.‐N.Z., Y.G., and X.‐Y.X. All authors have read and agreed to the published version of the manuscript.

## Ethics Statement

There were no experiments dealing with animal or human subjects or tissue samples from human subjects in the study.

## Conflicts of Interest

Hongbo Zhang is an executive editor for *Smart Medicine* and was not involved in the editorial review or the decision to publish this article. All authors declare that there are no competing interests.

## Supporting information

Supporting Information S1
