# Supplementary material for: Biomineralize Mitochondria in Metal‐Organic Frameworks to Promote Mitochondria Transplantation From Non‐Tumorigenic Cells Into Cancer Cells
Source: Smart Med. 2025 Feb 26;4(1):e134. doi: 10.1002/smmd.134 (PMC11862567; doi:10.1002/smmd.134)
Supplement: Supplementary file 1 — Supporting Information S1 [file SMMD-4-e134-s001.docx]

Supporting Information

Biomineralize Mitochondria in Metal-Organic Frameworks to Promote Mitochondria Transplantation from Non-Tumorigenic Cells into Cancer Cells

Jun-Nian Zhou^†,^*, Chang Liu^†^, Yong-Hui Wang^†^, Yong Guo, Xiao-Yu Xu, Elina Vuorimaa-Laukkanen, Oliver Koivisto, Anne M. Filppula, Jiangbin Ye*, Hongbo Zhang*

J.N. Zhou, C. Liu, Y.H. Wang, Y. Guo, X.Y. Xu, Oliver Koivisto, A.M. Filppula, H. Zhang

Pharmaceutical Sciences Laboratory, Faculty of Science and Engineering, Åbo Akademi University, Turku 20520, Finland

J.N. Zhou, C. Liu, Y.H. Wang, Y. Guo, X.Y. Xu, H. Zhang

Turku Bioscience Centre, University of Turku and Åbo Akademi University, Turku 20520, Finland

J. Ye

Department of Radiation Oncology, Stanford University School of Medicine, Stanford, California, USA

J.N. Zhou

Beijing Institute of Radiation Medicine, Beijing 100850, China

1. Guo

Department of Endocrinology, Key Laboratory of National Health & Family Planning Commission for Male Reproductive Health, National Research Institute for Family Planning, Beijing 100081, China

Elina Vuorimaa-Laukkanen,

Chemistry and Advanced Materials, Faculty of Engineering and Natural Sciences, Tampere University

† These authors contributed equally to this work

* Correspondence: hongbo.zhang@abo.fi (H. Zhang), zhoujunnian@bmi.ac.cn (J.N. Zhou) and yej1@stanford.edu (J.B. Ye)

**Supporting Figures**

**
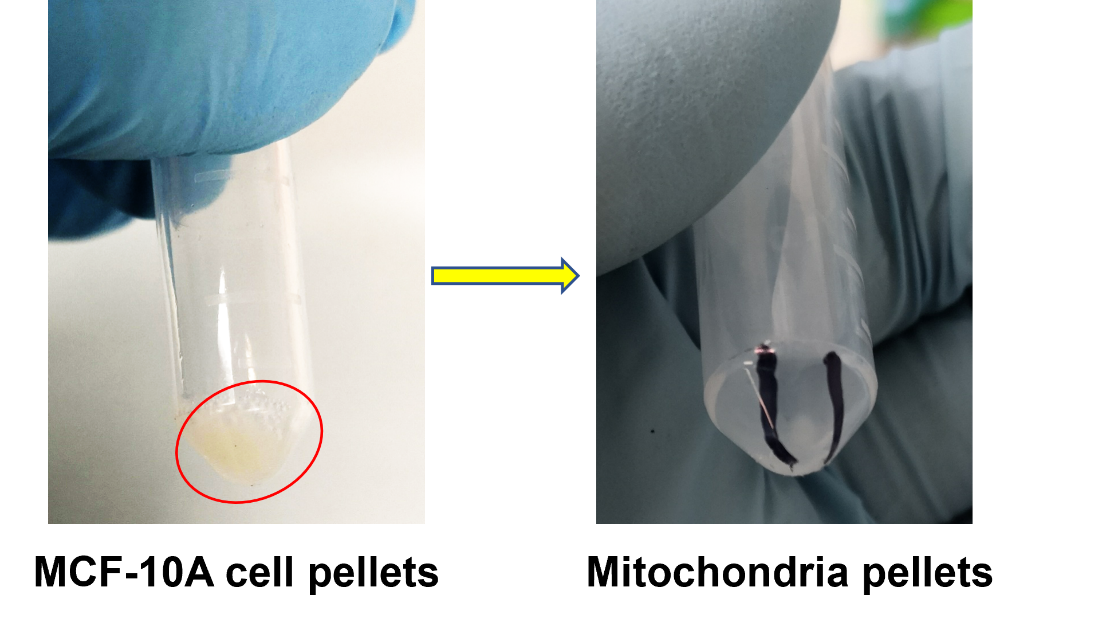
**

**Figure S1. Isolation of mitochondria from cultured human mammary epithelial cells MCF-10A.**

**
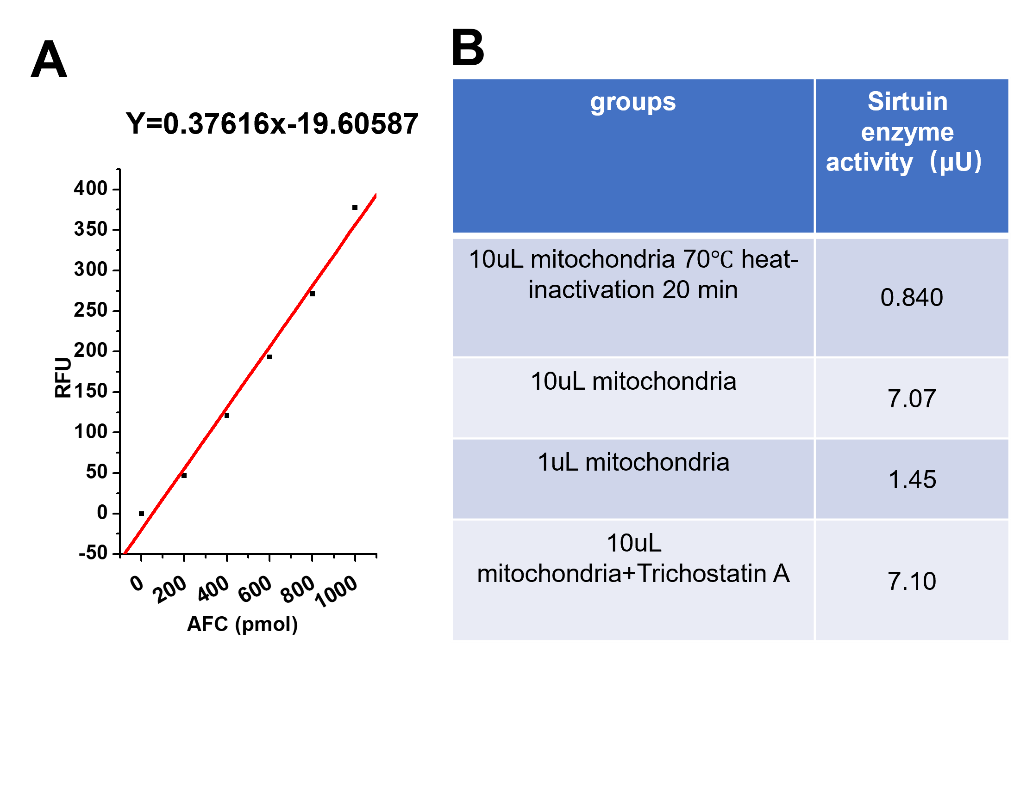
**

**Figure S2. Sirtuin enzyme activity measurement of freshly isolated mitochondria.** (A) The standard AFC standard curve for sirtuin enzyme activity measurement. (B) Sirtuin enzyme activity measurement was detected fluorometrically at λ_ex_ = 400 nm/λ_em_ = 505 nm, using sirtuin activity assay kit (Fluorometric, Sigma-Aldrich). According to the manufacturer’s instructions, Trichostatin A was added to the reaction to specifically inhibit HDACs which will also deacetylate the acetylated p53-AFC substrate to produce fluorescent group.


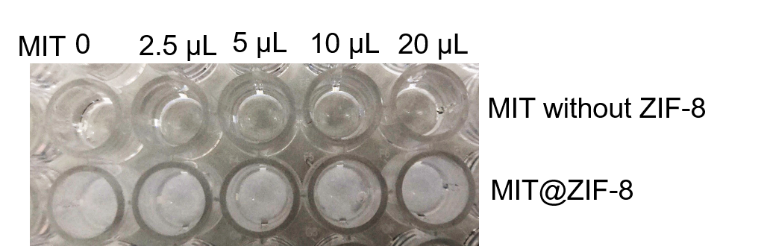


**Figure** **S3.** **Gross observation after formation of ZIF-8-mitochondrial nanocomplex.** Generation of white nanocomposites after one-pot synthesis within 5 min.

**
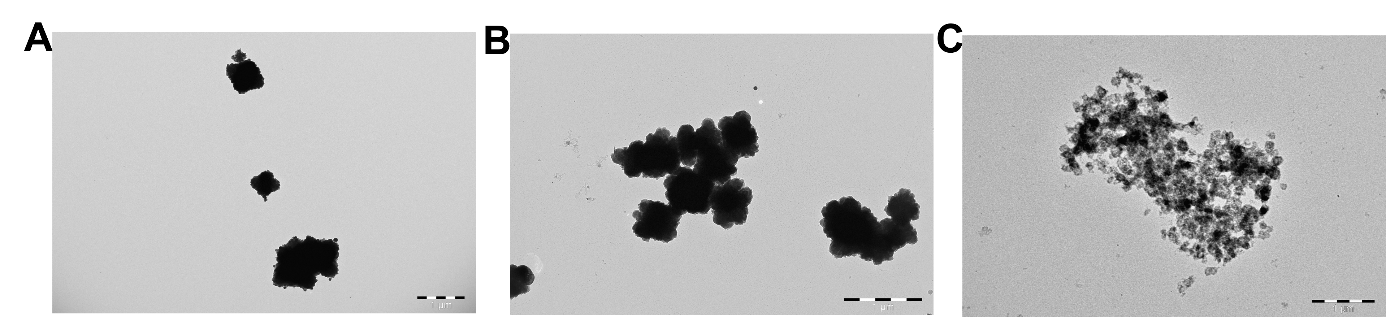
**

**Figure S4. 0.9% NaCl solution was selected for mixed reaction of ZIF-8 coating mitochondria and storage.** (A) TEM images of null ZIF-8 NPs (0.9% NaCl solution). (B) TEM images of ZIF-8-coated mitochondria (0.9% NaCl solution). (C) TEM images of ZIF-8-coated mitochondria (PBS solution). Scale bars, 1 μm.


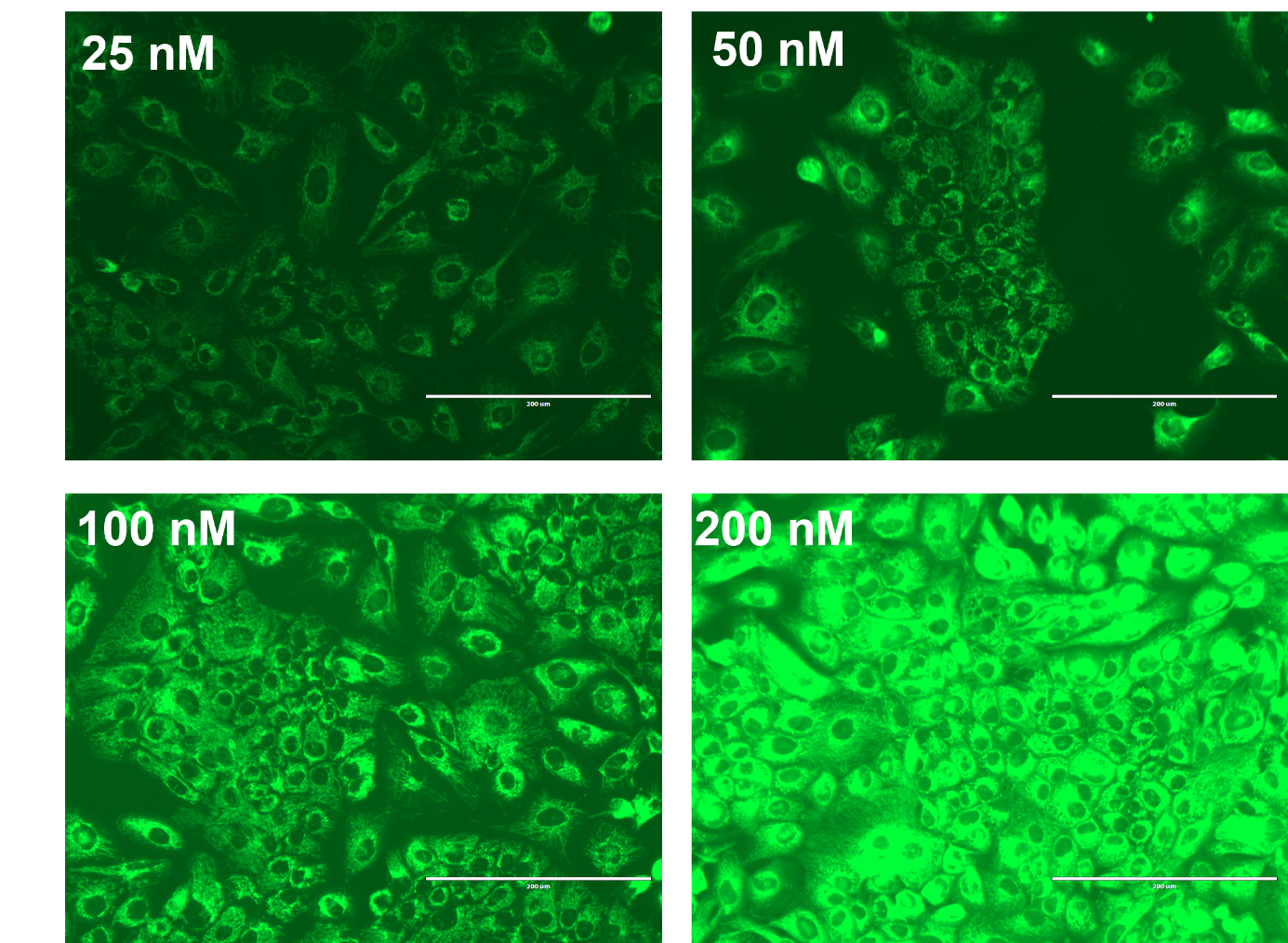


**Figure S5. Concentration series of Mitoview Green (MVG) staining mitochondria overnight in MCF-10A cells, observed under fluorescence microscopy.** Scale bar, 200 μm.

**
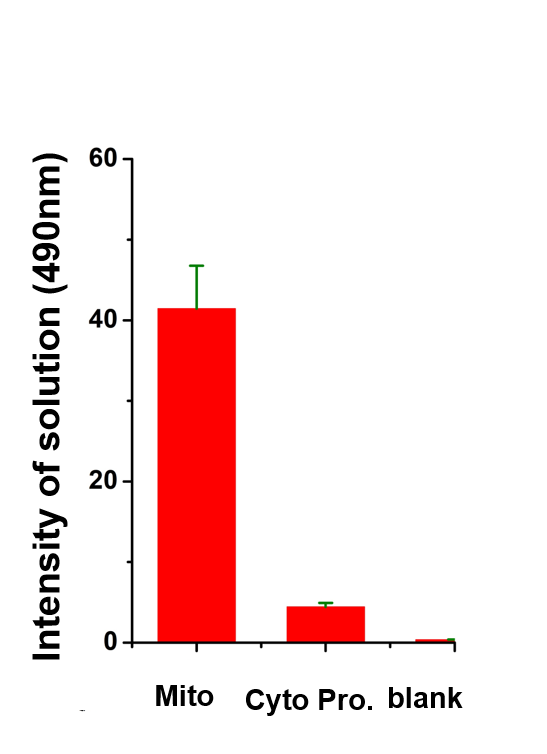
**

**Figure S6. Fluorescence intensity of isolated mitochondria (mito) and cytoplasm protein (Cyto Pro.) from MVG dye-labeled MCF-10A cells by mitochondria isolation kit.**

**
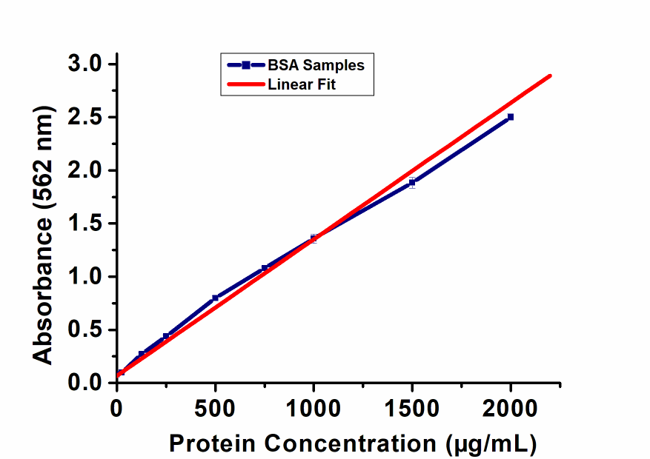
**

**Figure S7. The standard curve for BCA assay with BSA samples.**

**
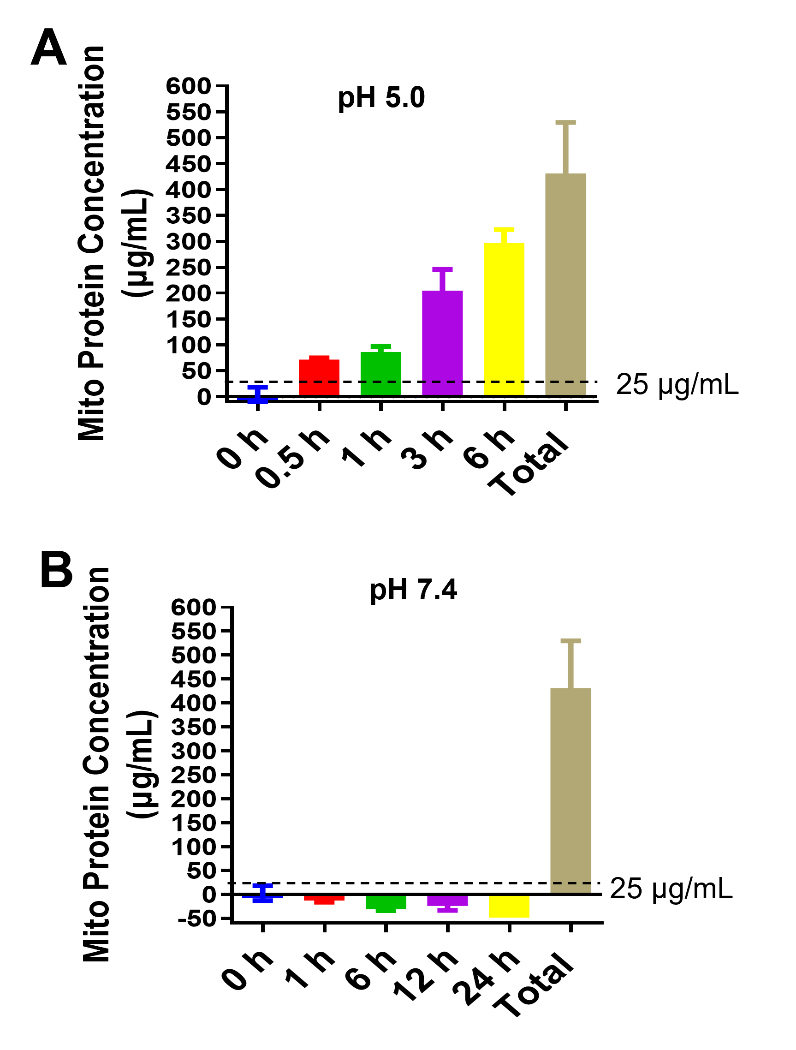
**

**Figure S8. Mitochondrial protein concentration in release solution at different time points by BCA assay.**

**
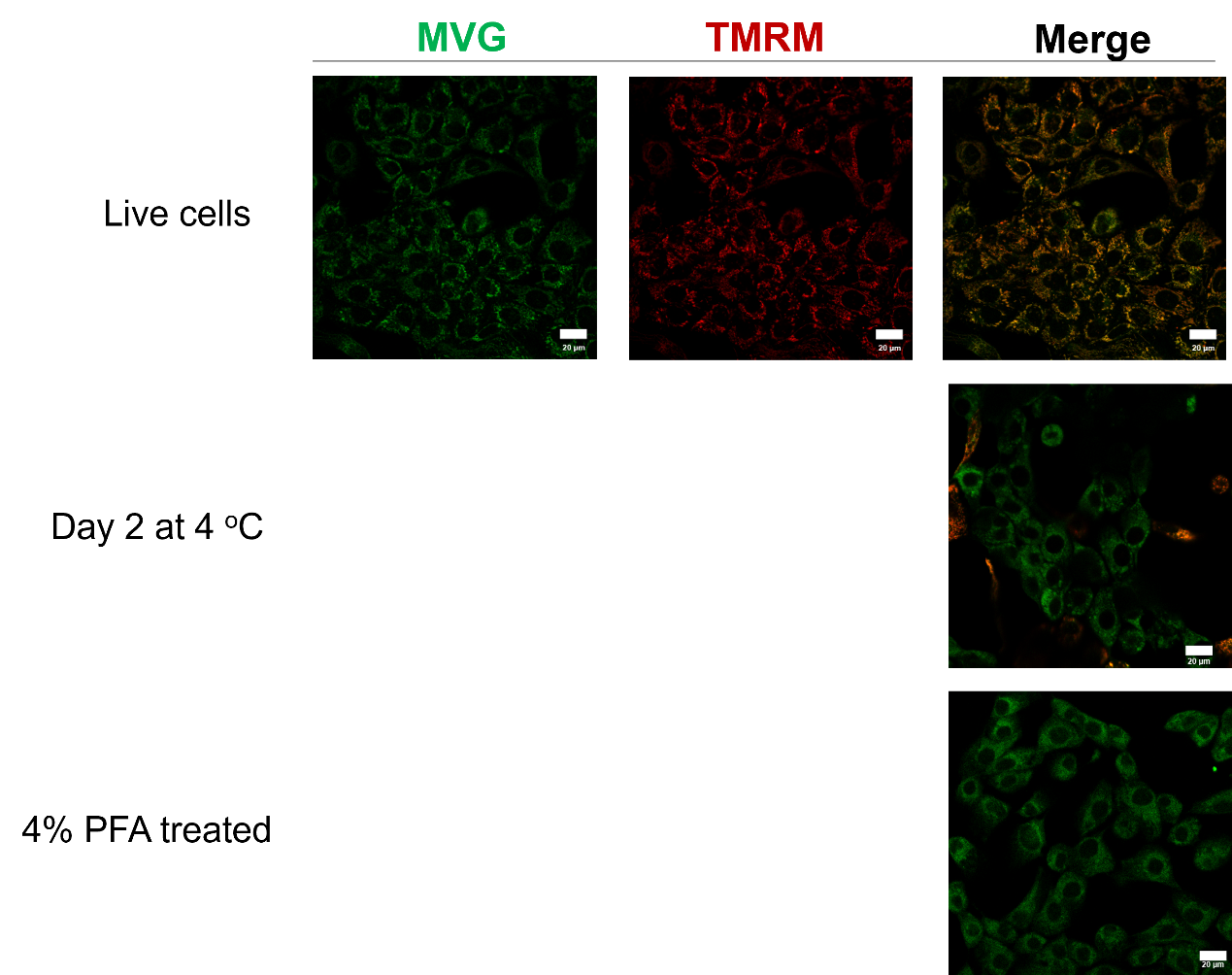
**

**Figure S9. CLSM images of TMRM and MVG dual labeled MCF-10A cells under different conditions.** Scale bars, 20 μm.


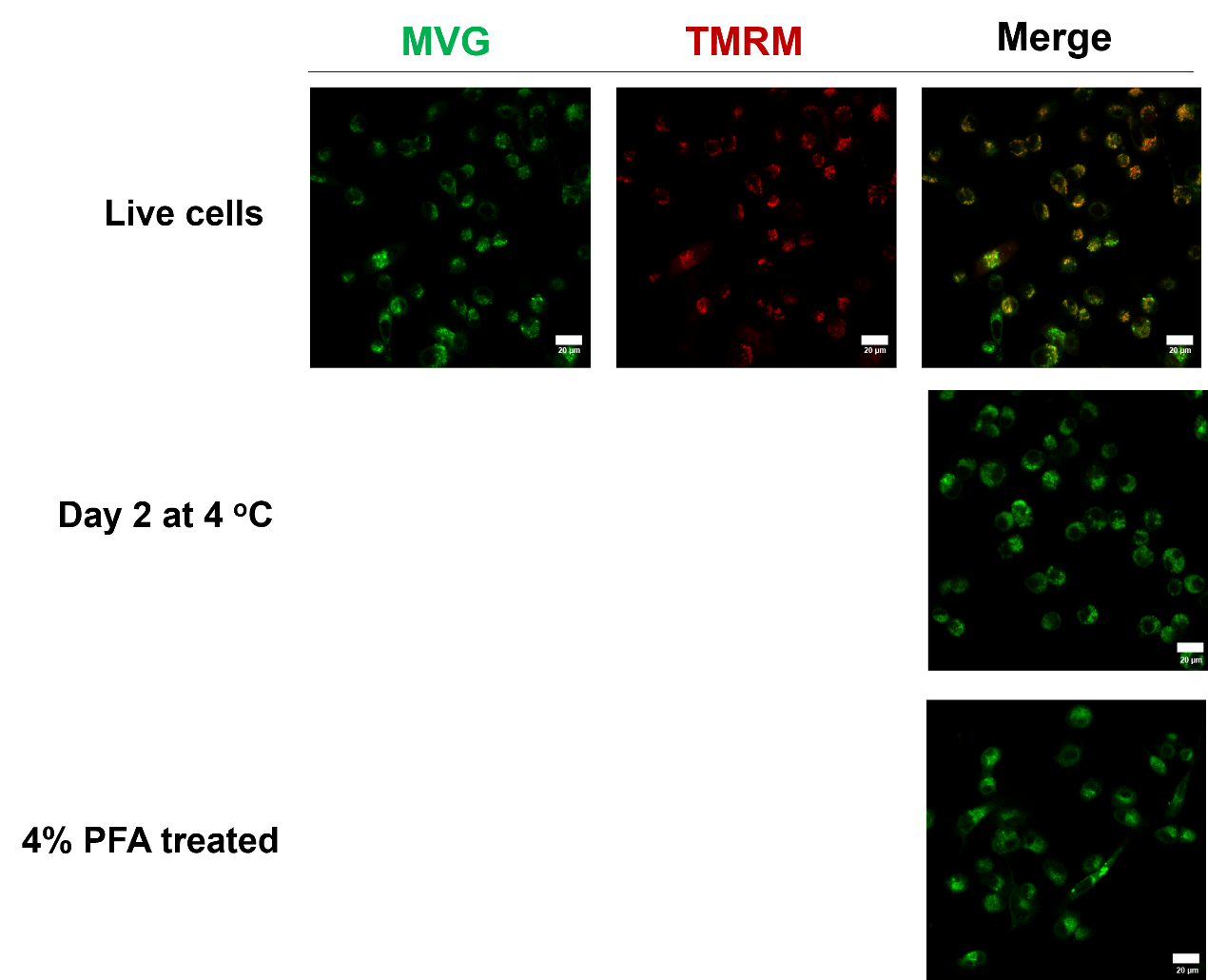


**Figure S10. CLSM images of TMRM and MVG dual labeled MDA-MB-231 cells under different conditions.** Scale bars, 20 μm.


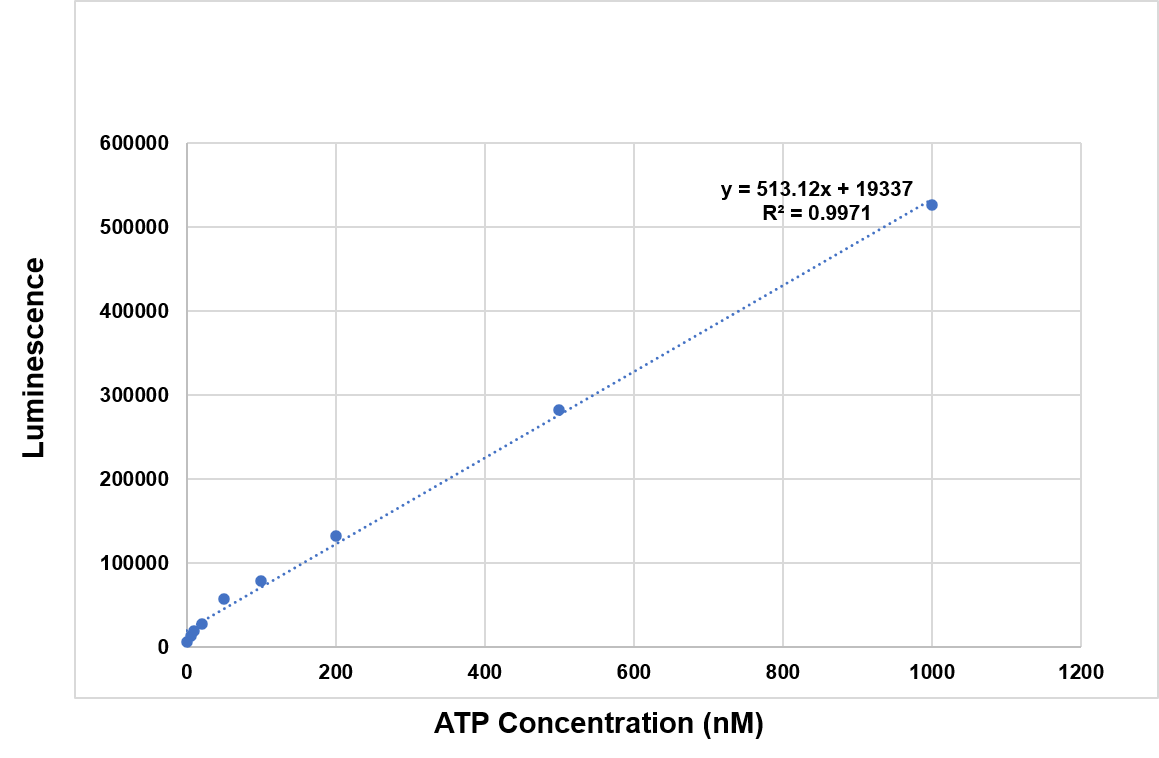
**Figure S11. The standard curve for ATP concentration.**

**Table S1 Fluorescence lifetimes, amplitudes of the longer living component and average lifetimes from the two-exponential fits of the MVG decay curves in different samples.**

| **Sample** | **t_1_ (ns)** | **t_2_ (ns)** | **a_2_ %** | **<t> (ns)** |
| --- | --- | --- | --- | --- |
| MVG-MIT | 0.23 ± 0.02 | 1.50 ± 0.04 | 29 | 0.60 |
| MVG-MIT@ZIF-8 | 0.27 ± 0.05 | 1.79 ± 0.04 | 55 | 1.11 |

**Table S2 The antibodies used in the study.**

| **Name** | **Catalogue number** | **Source** | **Dilution** |
| --- | --- | --- | --- |
| Vimentin | 16362885 | ThermoFisher Scientific | 1:1000 |
| E-cadherin | 16362035 | ThermoFisher Scientific | 1:1000 |
| Snail 1 | 17228723 | ThermoFisher Scientific | 1:1000 |
| N-cadherin | 17219483 | ThermoFisher Scientific | 1:1000 |
| GAPDH | 97166 | Cell Signaling Technology | 1:1000 |
| β-actin | 3700 | Cell Signaling Technology | 1:1000 |
| Anti-rabbit IgG, HRP-linked Antibody | 7074 | Cell Signaling Technology | 1:1000 |
| Anti-mouse IgG, HRP-linked Antibody | 7076 | Cell Signaling Technology | 1:1000 |
| CD44 Monoclonal Antibody (IM7), APC | 17-0441-82 | eBioscience | 0.15 μL/test |
| CD24 Monoclonal Antibody (eBioSN3 (SN3 A5-2H10)), PE | 12-0247-42 | eBioscience | 5 μL/test |
| Mouse IgG1 kappa Isotype Control (P3.6.2.8.1), APC | 17-4714-42 | eBioscience | 0.3 μL/test |
| Mouse IgG1 kappa Isotype Control (P3.6.2.8.1), PE | 12-4714-81 | eBioscience | 1.25 μL/test |
